# Unsuspected diversity of *Niphargus *amphipods in the chemoautotrophic cave ecosystem of Frasassi, central Italy

**DOI:** 10.1186/1471-2148-10-171

**Published:** 2010-06-09

**Authors:** Jean-François Flot, Gert Wörheide, Sharmishtha Dattagupta

**Affiliations:** 1 Courant Research Centre "Geobiology", University of Göttingen, 37077 Göttingen, Germany; 2 Department für Geo- und Umweltwissenschaften & GeoBio-CenterLMU, Ludwig-Maximilians-Universität München, 80333 München, Germany

## Abstract

**Background:**

The sulfide-rich Frasassi caves in central Italy contain a rare example of a freshwater ecosystem supported entirely by chemoautotrophy. *Niphargus ictus*, the sole amphipod species previously reported from this locality, was recently shown to host the first known case of a freshwater chemoautotrophic symbiosis. Since the habitat of *N. ictus *is highly fragmented and is comprised of streams and lakes with various sulfide concentrations, we conducted a detailed study to examine the potential genetic diversity of this species within Frasassi.

**Results:**

By sequencing one nuclear (ITS) and two mitochondrial (COI and 12S) regions, we show that four partially sympatric *Niphargus *clades are present in Frasassi. Morphological and behavioral data obtained for three of these clades are perfectly congruent with this molecular delineation and make it possible to distinguish them in the field. Phylogenetic analyses of 28S ribosomal DNA sequences reveal that, among the four clades, only two are closely related to each other. Moreover, these four clades occupy distinct niches that seem to be related to the chemical properties and flow regimes of the various water bodies within Frasassi.

**Conclusions:**

Our results suggest that four distinct *Niphargus *species are present in Frasassi and that they originated from three or four independent invasions of the cave system. At least two among the four species harbor *Thiothrix *epibionts, which paves the way for further studies of the specificity and evolutionary history of this symbiosis.

## Background

Groundwater ecosystems are under constant threat from anthropogenic activities, yet remain relatively understudied to date [1]. Amphipods are a major component of the fauna inhabiting these ecosystems[2] and the largest genus among them is *Niphargus*, which is distributed across most of Europe [3]. Like many other groups of subterranean metazoans, *Niphargus *amphipods are almost always blind [4] and usually white [5], hence their generic name (from the Greek word niphargês meaning "white like snow"). The taxonomy of the genus *Niphargus *has been debated for the last 160 years [6] and is presently in a state of flux with the discovery of numerous cryptic taxonomic units [7-9]. Thus, understanding the biodiversity of this genus is of crucial importance to better grasp the origin and dispersion of the European groundwater fauna and to guide conservation efforts, as has previously been highlighted for gammarid amphipods in other ecosystems [10,11].

Unlike subterranean ecosystems that are fed by aboveground photosynthetic productivity [12], chemoautotrophic caves such as Movile in Romania [13], Frasassi in Italy [14] and Ayyalon in Israel [15] receive their energy input mostly in the form of the chemical hydrogen sulfide arising from underground reservoirs. Chemoautotrophic microorganisms use the energy derived from sulfide oxidation to fix carbon and thereby form the basis of the food chain in these thriving cave ecosystems. However, sulfide is toxic for aerobic organisms [16,17] as it inhibits mitochondrial electron transport [18]; besides, it reacts with oxygen, causing hypoxia [19]. Hence, animals inhabiting sulfide-rich environments (such as marine sediments [20], hydrothermal vents [21], anchihaline caves [22] and sulfidic caves [23]) face specific metabolic challenges that they counter with avoidance behaviors, adaptations such as sulfide-oxidizing mitochondria and sulfide-binding proteins, or symbioses with sulfide-oxidizing bacteria [24].

Amphipods generally have a low tolerance to sulfide, even though some species may be quite resistant to hypoxia [25-28]. *Niphargus ictus *is the numerically dominant macroorganism in the sulfide-rich Frasassi cave ecosystem and has been the sole amphipod species reported to date from this location [14,29,30]. It thrives in the sulfidic streams and pools found in various parts of the cave, and a possible explanation for its tolerance to sulfide may lie in its symbiosis with chemoautotrophic sulfur-oxidizing bacteria of the genus *Thiothrix *[31]. Such chemoautotrophic symbioses are common in marine environments [32], but appear much rarer in freshwater where the *N. ictus *symbiosis is the only example reported to date.

Caves in Frasassi are developed in a limestone platform interspersed with a network of fractures that influence its hydrogeology [14] and could cause habitat fragmentation. Furthermore, the streams and lakes within the cave system have widely different sulfide and oxygen concentrations [33], which could lead to the occurrence of distinct *Niphargus *populations with varying tolerances to sulfide and hypoxia. To test these hypotheses, we sequenced mitochondrial and nuclear sequence markers for 184 *Niphargus *samples collected throughout the accessible parts of the cave system and complemented these molecular analyses with morphological and behavioral observations. Since our study unexpectedly revealed the presence of four *Niphargus *clades within Frasassi, we further examined the relationship between these clades by building a 28S ribosomal DNA (rDNA) phylogeny including all *Niphargus *sequences presently available in GenBank.

## Results

### Molecular analyses

**Figure 1:**
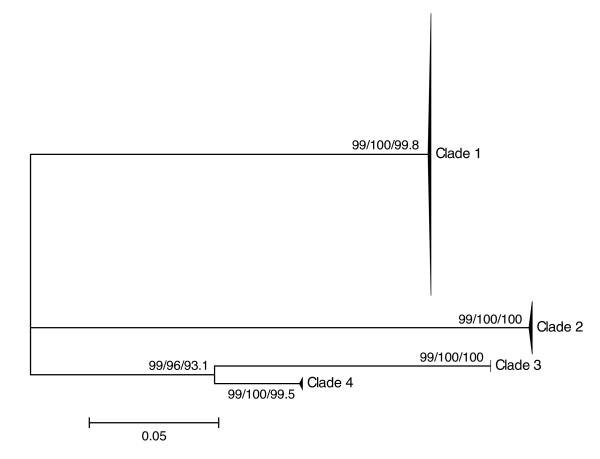
**Unrooted maximum-likelihood tree of 12S mitochondrial sequences**. This tree was generated with PhyML under the model TIM3+G (127 parameters) selected by jModelTest. Individual sample names are not shown for the sake of clarity, and Neighbor-joining (NJ)/Maximum Parsimony (MP)/Maximum Likelihood (ML) bootstrap values (1000 replicates) are displayed next to each node.

**Figure 2:**
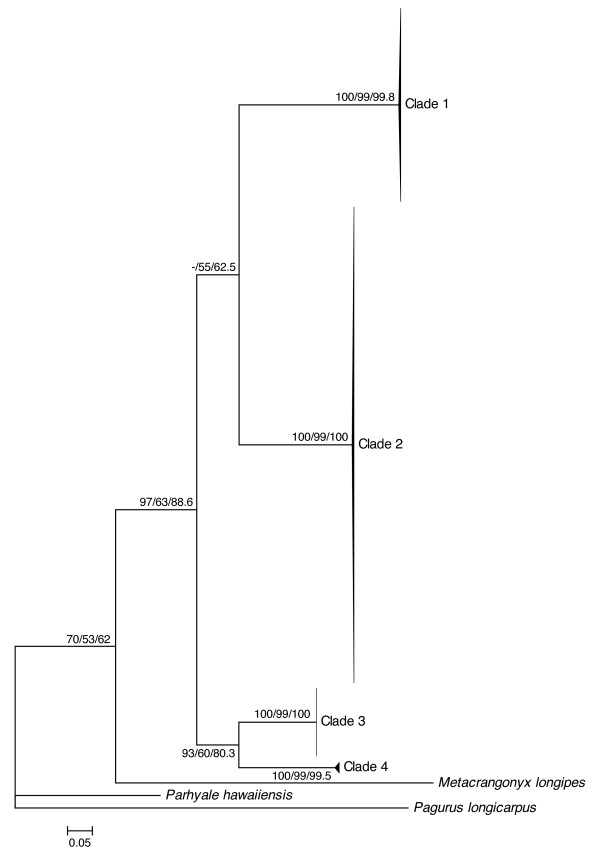
**Rooted maximum-likelihood tree of COI mitochondrial sequences**. This tree was generated with PhyML under the model TIM1+I+G (80 parameters) selected by jModelTest. Individual sample names are not shown for the sake of clarity, and NJ/MP/ML bootstrap values (1000 replicates) are displayed next to each node.

**Figure 3:**
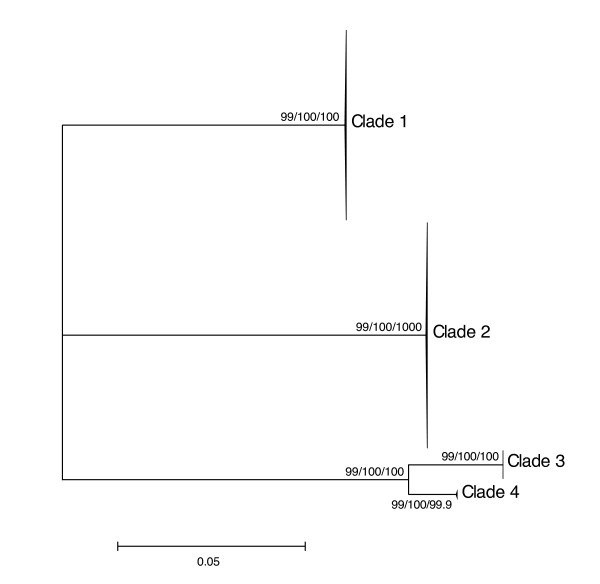
**Unrooted maximum-likelihood tree of ITS nuclear sequences**. This tree was generated with PhyML under the model TVM+G (40 parameters) selected by jModelTest. Individual sample names are not shown for the sake of clarity, and NJ/MP/ML bootstrap values (1000 replicates) are displayed next to each node.

**Figure 4:**
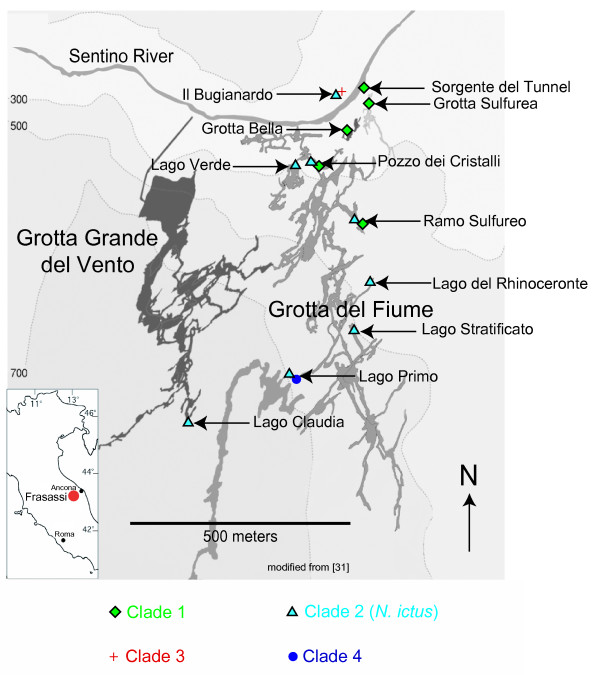
**Map of the Frasassi cave system showing the observed occurrences of each species**. All natural and man-made cave entrances are located in the vicinity of the Sentino River. Clade 1 (green diamonds) is only found in the northeastern part of the cave system, Clade 2 (blue triangles) is found nearly everywhere except in three locations in the northeast, Clade 3 (red cross) was only sampled in one site on the northern side of the Sentino river, and Clade 4 (blue circle) was only collected in one site in the south (Lago Primo).

For each marker analyzed (12S, ITS, COI), results obtained using distance, parsimony and likelihood methods were congruent in delimiting four *Niphargus *clades among our samples (Figures 1, 2 and 3). Clade 1 comprised 75 individuals collected in five sampling sites in the north-eastern part of the cave complex (Figure 4), Clade 2 grouped 94 samples from all sites except three (where only Clade 1 was present), Clade 3 comprised 13 specimens from a single location on the northern side of the river (Il Bugianardo), and Clade 4 was represented by only two individuals from one remote site in the south (Lago Primo). Bootstrap values for the monophyly of the clades were very high (>99) using all three phylogenetic methods. Some locations were sampled more than one time in two or three different years (Table 1) but no time variation in the geographical repartition of the clades was detected. Average patristic distances between clades calculated from the COI tree (Table 2) were all above the 0.16 threshold proposed for species delimitation in Crustacea [34]; the lowest value was found for the distance between Clades 3 and 4, indicating that these two clades are the most closely related among the four.

**Table 1:** Overview of the specimens analyzed (including a description of the sampling sites and their main geochemical parameters)

Sampling site (abbreviations)	Type of water body	**Geochemistry**^**a**^		Number of specimens analyzed (per collection year)		
		
		[H_2_S] (μM)	[O_2_] (μM)	2007	2008	2009
Il Bugianardo (BUG, BG)	Stagnant pool	n.d.	151		17	

Sorgente del Tunnel (ST)	Flowing stream	136	31			8

Grotta Sulfurea (GS)	Stream with stagnant eddies	118	51		14	2

Grotta Bella (GB)	Flowing stream	45	6		8	1

Pozzo dei Cristalli (PDC, PC)	Flowing stream with stagnant pond caused by obstruction	415	12	11	23	8

Lago Verde (LVE, LV)	Stagnant lake	415	2	10	23	

Ramo Sulfureo (RS)	Stream with stagnant eddies	109	10	7	18	

Lago del Rinoceronte (LRI)	Stagnant lake (stratified ^b^)	n.a	n.a.		8	

Lago Stratificato (LST)	Stagnant lake	n.a.	n.a.		5	

Lago Primo (LPR)	Stagnant lake (stratified ^b^)	n.a.	n.a.		6	

Lago Claudia (LCL, LC)	Stagnant lake	45	8		8	7

^a ^all measurements reported here were performed in May-June 2009

^b ^i.e., with a sulfidic lower layer and an oxygenated upper layer

n.d. = non detectable

n.a. = not available

**Table 2:** Average patristic distances within and between clades (computed from the COI maximum likelihood tree)

	Clade 1	Clade 2	Clade 3	Clade 4
Clade 1	0.0045			

Clade 2	0.5638	0.0032		

Clade 3	0.6561	0.5619	0.0004	

Clade 4	0.7021	0.6079	0.3596	0.0172

**Figure 5:**
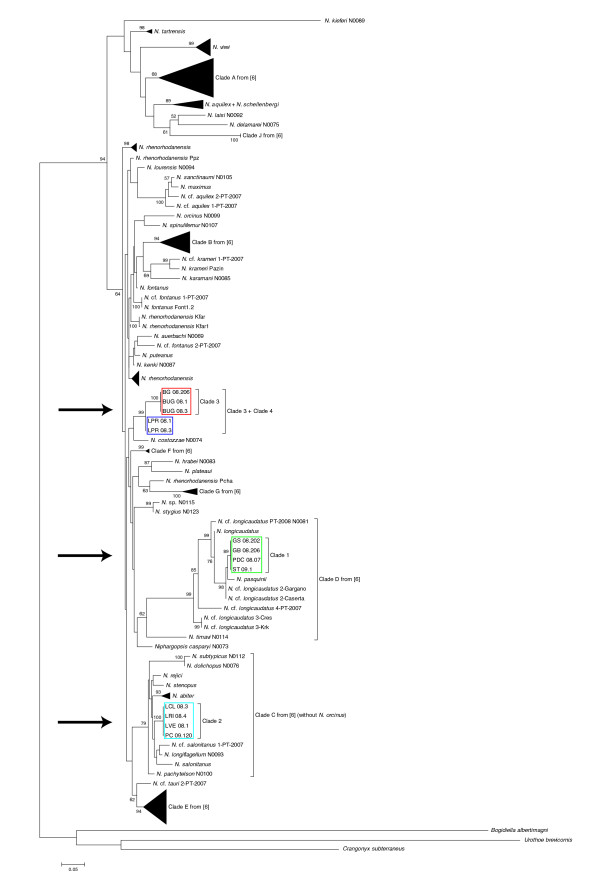
**Maximum-likelihood 28S phylogeny of the genus *Niphargus***. This tree was generated with PhyML under the model SYM+G (290 parameters) selected by jModelTest. For the sake of clarity, only ML bootstrap values higher than 50% are displayed and the long branches leading to *Bogidiella albertimagni*, *Crangonyx subterraneous*, *Urathoe brevicornis *and *Niphargus kieferi *are drawn with only half of their actual lengths. Clades 1-4 (arrows) are from the present study, Clades A-J are from [6].

A 28S rDNA phylogeny of the genus *Niphargus*, including representative sequences from each Frasassi clade and all sequences available in GenBank, confirmed the separation between the clades and the close relationship between Clades 3 and 4 (Figure 5). The *Niphargus *present in Frasassi do not form a monophyletic group within the genus but fall instead into three distinct regions of the tree.

### Morphological analyses

**Figure 6:**
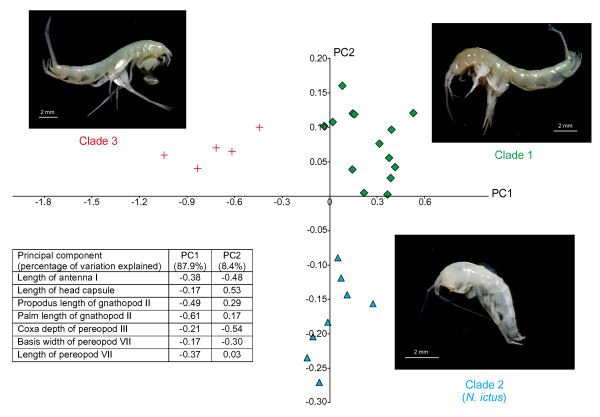
**Principal components analysis of seven quantitative morphological characters measured on 27 *Niphargus *individuals from Frasassi**. Insert shows the loadings of each individual character on the two principal components (green diamonds: Clade 1; blue triangles: Clade 2; red crosses: Clade 3)

The morphometric analysis of seven quantitative characters shows that Clades 1-3 are morphologically distinct, with no overlap among them (Figure 6). The first component (horizontal on the figure) explained 87.9% of the variation (eigenvalue: 0.164) and distinguished Clade 3 from the other two predominantly based on its larger gnathopods. The second component (vertical) explained 8.4% of the variation (eigenvalue: 0.016) and distinguished Clade 2 from the other two based on its smaller head, longer antennae and deeper ventral channel (located between the coxal plates and bases of pereopods V to VII [35]).

Only the morphology of Clade 2 individuals corresponds to the published description of *N. ictus *[30]: Clade 1 individuals seem related to *N. longicaudatus*, a complex of several cryptic species or subspecies in need of taxonomic revision [9], whereas Clade 3 shares some morphological characteristics with the *N. rejici *species complex found in Slovenia and some Adriatic islands [36]. As for Clade 4, for which only two badly damaged samples are available at the present time, its morphology appears similar to Clade 3 (also its closest relative in Frasassi according to our molecular data) but more specimens need to be collected before definite conclusions can be reached regarding its identity.

### Behavioral observations

The behavior of Clade 1 individuals was observed in two sites (Grotta Sulfurea and Sorgente del Tunnel), where they were found to spend most of their time crawling on their sides on bacterial mats and sediment. When disturbed, they swam poorly and for short durations (less than five seconds). In contrast, Clade 2 individuals observed in Lago Verde were found to be strong swimmers, achieving speeds of 1-2 body lengths per second, and were able to swim continuously for more than 10 seconds. In Pozzo dei Cristalli, a 10-meter long stream with a stagnant pool in the middle, some *Niphargus *were observed to crawl on bacterial mats whereas others were swimming in the stagnant pool. As our 2007 and 2008 data showed that both Clades 1 and 2 were present in this location, 4 individuals of each behavioral type were collected in 2009 and analyzed morphologically and molecularly: all crawling *Niphargus *were found to belong to Clade 1, whereas all swimming individuals belonged to Clade 2.

Even though Clade 3 was only present in Il Bugianardo where it co-occurred with Clade 2, it could be easily distinguished in the field due to its much larger size, allowing comparative behavioral observations of Clade 2 and Clade 3 individuals in this location. Clade 2 individuals were found to spend most of their time swimming in the deeper parts of the pool, whereas Clade 3 predominantly crawled on limestone boulders.

## Discussion

### Four *Niphargus *clades are present in Frasassi, among which only two are closely related to each other

Prior to this study, only one *Niphargus *species had been reported from the Frasassi cave system and this species, described as *N. ictus*, was supposed to be endemic to this locality [14,29]. Here we show that 4 molecularly distinct clades are actually present in Frasassi, with patristic distances (Table 2) between them higher than the 0.16 threshold proposed for species delimitation in Crustacea [34]. The perfect agreement between independent nuclear and mitochondrial datasets further suggests that these four clades are not just an instance of intraspecific variation but represent four distinct monophyletic taxa of putative species level. Among them, Clades 1-3 can be also be diagnosed morphologically and behaviorally (unfortunately, no intact representative of Clade 4 is presently available for morphological analyses, and the behavior of living individuals from this rare clade could not be observed). Moreover, there was no heterozygous individual harboring nuclear ITS sequences from more than one clade: thus, these four clades appear reproductively isolated from each other [37], which further suggests that they represent distinct species following the biological species criterion [38].

The most parsimonious explanation for the observed polyphyly of *Niphargus *within Frasassi (Figure 5) is that this cave system was colonized at least three times by different amphipod lineages since the start of its formation less than 1 million years ago [31]: one lineage that had only been reported until now from the Dinaric Region (Slovenia, Croatia, Herzegovina), a second lineage found presently on both sides of the Adriatic Sea (Italy, Croatia, Greece), and a third lineage (comprising Clades 3 and 4 from the present study) only found in Frasassi to date. Analysis of samples from caves in the surrounding area will be required to find out whether the closely related Clades 3 and 4 invaded the Frasassi cave system independently or if speciation occurred within Frasassi from their invading common ancestor.

### Water chemistry and flow regime seem to influence the repartition of the four clades in Frasassi

The areas of occurrence of the four clades in Frasassi are overlapping (Figure 4) but show some correlation with groundwater chemistry and hydrological flow regimes. Clade 1 occurs only in shallow, flowing streams and predominantly at relatively low sulfide concentrations (45 to 150 μM sulfide); however, sulfide tolerance does not appear to be a limiting factor for this clade since it is also found in Pozzo dei Cristalli, a stream characterized by much higher sulfide levels (up to 415 μM). Clade 2 (that corresponds to the published description of *N. ictus*) is predominantly found in lakes with calm and deep waters (>20 cm depth), with sulfide concentrations spanning the whole range of values measured in Frasassi (i.e., from non-detectable to 415 μM sulfide). Clade 3 occurs only in Il Bugianardo, a location with no detectable sulfide that is also the only sampling site located north of the Sentino River. Clade 4 has so far only been collected from Lago Primo, a stratified lake with oxygenated water on top and reducing, sulfidic waters at depths greater than 3.5 meters (JL Macalady, pers. comm.). At a more local scale, members of various clades were found to occupy different microhabitats in locations where they co-occur. In Pozzo dei Cristalli, where Clades 1 and 2 cohabitate, Clade 1 was restricted to fast-flowing portions of the stream that were less than 5 cm deep and Clade 2 to stagnant parts that were deeper than 20 cm. In Il Bugianardo, Clades 2 and 3 co-occur in a small pool that is 10- 50 cm deep: there, Clade 3 members were found crawling on limestone boulders and within interstitial crevices, whereas Clade 2 members were swimming in the deeper parts of the lake.

The "crawling" species (Clades 1 and 3) have restricted areas of repartition in comparison with the "swimming" Clade 2 (*N. ictus*) that was sampled everywhere except in three streams (Figure 4). Large lakes with deep water dominate the southern recesses of the Frasassi cave network, and a gradual slope causes water to flow towards the Sentino River. The remarkable swimming ability of *N. ictus *could enable it to move upstream and across large bodies of deep water, allowing it to occupy most of the water bodies within Frasassi (the question of whether *N. ictus *populations on both sides of the river are connected will require future work using highly variable markers such as microsatellites). Clade 1, on the other hand, might be restricted by its poor swimming ability to shallow streams (found only in northern parts of the cave system close to the Sentino River), whereas the limited distribution of Clade 3 may be either due to its inability to cross the barrier created by the Sentino River or to its lower tolerance to sulfide compared with Clades 1 and 2. Unlike Clade 2 (*N. ictus*) that is found in most parts of the cave system (including deep recesses rarely visited by cavers), Clades 1 and 3 appear restricted to few, easy-to-reach locations and may require specific conservation efforts in the future.

### At least two of the Frasassi *Niphargus *clades are symbiotic

When the symbiosis between *Niphargus *and sulfur-oxidizing bacteria was discovered in Frasassi [31], all the *Niphargus *individuals used in that study were assumed to be conspecific since *N. ictus *was the only species reported within the cave system [14,29]. The unexpected finding of four distinct clades in the present study prompted us to reexamine the previously published data regarding this symbiosis. Among these data, *Niphargus *individuals from the study sites Grotta Sulfurea, where only Clade 1 has been found, and Lago Verde, inhabited only by Clade 2 (*N. ictus*), were both found to be symbiotic with sulfur-oxidizing *Thiothrix *bacteria using fluorescence *in situ *hybridization (FISH) analyses [31]. We have subsequently confirmed this finding by scanning electron microscopy and by sequencing bacterial 16S rDNA libraries (unpublished results). Additional investigations will be required to find out whether Clades 3 and 4 are also symbiotic, and whether bacterial symbionts are different between *Niphargus *clades.

### COI and ITS are useful molecular markers for the taxonomy of *Niphargus*

Prior to the present study, the COI barcode marker [39] had only been sequenced for very few species of *Niphargus *[7,8] whereas the ITS region of this genus had never been analyzed. Here we demonstrate that COI and ITS can conjointly be used to delineate *Niphargus *species, and may become of standard use in future taxonomic studies of *Niphargus *as they are already the most commonly used markers in other groups of organisms [40]. The 12S marker, on the other hand, is shorter and more difficult to align than COI, but can still prove useful when dealing with populations or individuals for which obtaining non-ambiguous COI sequences is difficult (which was the case here with our Clade 1, see Materials and Methods). As for nuclear markers, an aim in future studies should be to develop primers targeting variable regions other than ITS, such as introns, as it is only by constructing and comparing several independent gene trees that one may hope to build a species tree with a reasonable degree of confidence [41-43].

## Conclusions

The molecular, morphological and behavioral data gathered in this study indicate that, instead of a single *Niphargus *species, the chemoautotrophic cave ecosystem of Frasassi hosts four distinct amphipod clades among which only two are closely related to each other; hence, there appears to have been at least three independent invasions of the Frasassi cave system. Moreover, the finding that two distantly related *Niphargus *clades harbor symbiotic *Thiothrix *bacteria paves the way for further studies concerning the origin and evolution of this unusual freshwater chemoautotrophic symbiosis.

## Methods

### Sample collection and processing

A total of 184 *Niphargus *individuals were collected in the Grotta Grande del Vento - Grotta del Fiume (Frasassi) cave system in 2007, 2008 and 2009 (Table 1) using tweezers or nets and preserved in 70% ethanol or RNAlater^® ^(Ambion). Most individuals were stored in single tubes, except for some population samplings where several individuals were collected together in a single large vial; even in such cases, we never encountered any evidence of DNA cross-contamination between our samples. Early DNA extractions were performed using one half of each individual; subsequently, only two appendages (one gnathopod and one pereiopod) from each individual were used for DNA extraction. All DNA extractions were performed using DNeasy^® ^Blood & Tissue kits (Qiagen) following the manufacturer's instructions.

### PCR amplification and sequencing

Partial COI (mitochondrial), 12S (mitochondrial) and 28S (nuclear) sequences were obtained using published primers (Table 3). PCR reactions (25 μL) were obtained by mixing 16 μL H_2_0, 2.5 μL PCR buffer, 1.3 μL DMSO, 1 μL MgCl_2 _(50 mM), 0.5 μL dNTP Mix (40 mM total), 0.3 μL each primer (25 mM), 3 μL DNA extract and 0.15 μL BIOTAQ^® ^(Bioline) in 200 μL Eppendorf tubes; PCR conditions consisted of 3 min at 94°C, then 50 cycles of 30 sec at 94°C, 1 min at 45°C and 1 min at 72°C. Two new primers (Table 3) were designed with the help of Primer3 [44] based on published amphipod 18S [45,46] and 28S [6-8] sequences, and used to amplify the complete ITS region (together with flanking portions of the 18S and 28S genes) by mixing 16.8 μL H_2_0, 2.5 μL Red Taq buffer, 1.3 μL DMSO, 0.5 μL dNTP Mix (40 mM total), 0.3 μL each primer (25 mM), 3 μL DNA extract and 0.6 μL Red Taq (Sigma) in 200 μL Eppendorf tubes; PCR conditions for this marker consisted of 1 min at 94°C, then 50 cycles of 30 sec at 94°C, 30 sec at 53°C and 3 min at 72°C.

**Table 3:** List of the primers used in this study

Marker	Direction	Purpose	Sequence	Reference
COI	Forward	PCR + sequencing	5'-GGTCAACAAATCATAAAGATATTGG-3'	[66]

COI	Reverse	PCR + sequencing	5'-TAAACTTCAGGGTGACCAAAAAATCA-3'	[66]

12S	Forward	PCR + sequencing	5'-GCCAGCAGCCGCGGTTA-3'	[67]

12S	Reverse	PCR + sequencing	5'-CCTACTTTGTTACGACTTAT-3'	[67]

28S	Forward	PCR + sequencing	5'-CAAGTACCGTGAGGGAAAGTT-3'	[68]

28S	Reverse	PCR + sequencing	5'-AGGGAAACTTCGGAGGGAACC-3'	[68]

28S	Forward	sequencing	5'-AAACACGGGCCAAGGAGTAT-3'	this article

28S	Reverse	sequencing	5'-TATACTCCTTGGCCCGTGTT-3'	this article

ITS	Forward	PCR + sequencing	5'-TCCGAACTGGTGCACTTAGA-3'	this article

ITS	Reverse	PCR + sequencing	5'-TCCAAGCTCCATTGGCTTAT-3'	this article

ITS	Forward	sequencing	5'-CGCTGCCATTCTCACACTTA-3'	this article

ITS	Reverse	sequencing	5'-ACTCTGAGCGGTGGATCACT-3'	this article

ITS	Forward	sequencing	5'-AAGGCTATAGCTGGCGATCA-3'	this article

ITS	Reverse	sequencing	5'-TCAGCGGGTAACCTCTCCTA-3'	this article

PCR products were cleaned using the QIAquick^® ^PCR Purification kit (Qiagen) and sequencing was performed using the same primers as for amplification. End-based sequencing turned out to be sufficient for the COI and 12S markers due to their relatively short lengths (658 bp and 477-480 bp, respectively). The 28S and ITS markers, however, were much longer (981-993 bp and 1589-2100 bp, respectively) and additional sequencing was performed using internal primers (Table 3).

### Molecular data analyses

Chromatograms were inspected, assembled and cleaned using Sequencher 4 (Gene Codes). ITS sequencing yielded a single, homozygous sequence for 164 individuals out of 184: as for the others, the chromatograms of 17 individuals comprised one double peak (i.e., these individuals possessed two nearly identical sequence types differing by one base), one individual had two double peaks, and two others had numerous double peaks (as expected when simultaneously sequencing two sequence types of different lengths [47]). Obtaining sequences was trivial in the first case, whereas the second case was solved using Clark's method [48] and the third case was phased using CHAMPURU [49]. For individuals comprising two distinct ITS types, sequence types were given the name of the individual followed by "a" or "b" and were all included in subsequent analyses. In order to allow comparison with *Niphargus *sequences present in GenBank, an additional 28S fragment was sequenced from 13 individuals representative of the four clades obtained using ITS (only one 28S haplotype was found in each sample analyzed).

We attempted to sequence the COI reference barcode fragment of all 184 individuals; however, the COI sequences obtained from 5 samples turned out to be of bacterial origin, whereas the chromatograms of 36 other individuals contained double or triple peaks indicating a mixture of several sequences and could not be interpreted unambiguously. Therefore, the COI sequences of only 143 *Niphargus *specimens out of 184 were used in further analyses. In order to ascertain the mitochondrial background of the remaining individuals, we resorted to sequencing 12S as a second marker: none of the samples for which COI sequencing had yielded double or triple peaks had more than one 12S sequence, whereas one individual that had a single COI sequence was found to possess two divergent 12S haplotypes (the sequences from this individual were discarded from subsequent analyses). Our final 12S dataset comprised 61 sequences.

All individuals that had double or triple peaks in their COI chromatograms belonged to Clade 1. The most likely explanation for the presence of multiple peaks is that one or several nuclear-transferred COI pseudogenes [50,51] were sequenced together with the mitochondrial COI; heteroplasmy, i.e. the occurrence of several distinct mitochondrial lineages within one individual, is a less probable explanation since there was no multiple peaks in the 12S chromatograms obtained from these individuals. In the same way, the presence of two very divergent 12S sequences in one individual from Clade 2 that had a single COI sequence strongly suggests that one of the two 12S sequences is a nuclear pseudogene.

All sequences obtained were deposited in public databases [GenBank: GU973003-GU973423]. COI sequences of the hermit crab *Pagurus longicarpus *and of the gammarid amphipods *Parhyale hawaiiensis *and *Metacrangonyx longipes *were extracted from their published complete mitochondrial genomes [52-54] and used to root the COI tree, whereas sequences from the amphipods *Bogidiella albertimagni*, *Crangonyx subterraneus *and *Urothoe brevicornis *were obtained from GenBank in order to root the 28S phylogeny. The ITS and 12S trees were left unrooted as *Niphargus *sequences for these markers were markedly divergent from their closest available relatives.

Sequences were aligned manually in MEGA4 [55] for COI and using MAAFT's Q-INS-I option [56,57] for ITS, 12S and 28S. The best-suited nucleotide model for each alignment was determined among 88 possible models following the Akaike Information Criterion [58] and the Bayesian Information Criterion [59] (whenever the two criteria disagreed, the more parameter-rich model was selected) as implemented in jModelTest [60], and used to build Maximum Likelihood (ML) phylogenetic trees with PhyML 3.0 [61]. In order to investigate the sensitivity of the results to variations in methods of phylogenetic reconstruction, Neighbor-Joining (NJ; nucleotide model: Kimura 2-parameter, uniform rates among sites, pairwise deletion) and Maximum Parsimony (MP; search options: CNI level = 1, initial tree by random addition with 10 replications, use all sites) analyses were also conducted on the same alignments using MEGA4. Furthermore, the robustness of the nodes obtained using each method was estimated by performing 1,000 bootstrap replicates [62]. Patristic distances were computed from the COI ML tree using the program PATRISTIC [63].

### Morphological analyses

A set of 27 ethanol-preserved samples from Il Bugianardo, Grotta Sulfurea, Pozzo dei Cristalli, Lago Verde, Ramo Sulfureo (5 individuals from each location) and Lago Primo (2 individuals), including both females and males but no juveniles, was examined for seven quantitative morphological characters that had previously proved useful for taxonomic investigation of *Niphargus *amphipods [64]: the length of the 1^st ^antenna, the length of the head capsule, the length of the propodus of the 2^nd ^gnathopod, the length of the palm of the 2^nd ^gnathopod, the depth of the coxal plate of the 3^rd ^pereopod, the width of the basal article of the 7^th ^pereopod and the length of the 7^th ^pereopod. Among the 27 samples, 14 turned out to belong to Clade 1, 8 to Clade 2 and 5 to Clade 3 (unfortunately, none of the two specimens from Clade 4 collected to date was intact enough to be included in this morphological analysis). *Niphargus *specimens were partially dissected in glycerin and mounted on slides. Appendages were photographed with an Olympus camera ColorView III mounted on a stereomicroscope Olympus DP Soft and measured using the program ANALYSIS (Olympus Soft Imaging Solutions). Principal components analysis was performed on the covariance matrix of the log-transformed measurements using the program PAST [65].

### Behavioral observations

Observations were conducted in May-June 2009 on about 200 individuals in five locations within the cave system: Il Bugianardo (Clades 2 and 3), Sorgente del Tunnel (Clade 1), Grotta Sulfurea (Clade 1), Pozzo dei Cristalli (Clades 1 and 2) and Lago Verde (Clade 2). Unfortunately the behavior of Clade 4 individuals could not be observed as they are found in a location (Lago Primo) that only very experienced cavers can access. We noted down the behavior of many individuals among the ones that we could see (since no attempt was made to mark them, some individuals were probably observed several times). When *Niphargus *amphipods did not swim spontaneously, we disturbed them by probing them with a pipette in order to document their swimming abilities. Digital photographs and videos were also taken whenever possible.

## Authors' contributions

JFF participated in fieldwork, designed primers, performed molecular labwork and computational analyses, conducted behavioral observations and drafted the manuscript. GW participated in initiating the study and revised the manuscript. SD designed and supervised the study, collected samples and geochemical data, conducted behavioral observations and revised the manuscript. All authors read and approved the final manuscript.
